# Blood pressure patterns in relation to geographic area of residence: a cross-sectional study of adolescents in Kogi state, Nigeria

**DOI:** 10.1186/1471-2458-8-411

**Published:** 2008-12-16

**Authors:** Chukwunonso ECC Ejike, Chidi E Ugwu, Lawrence US Ezeanyika, Ayo T Olayemi

**Affiliations:** 1 Department of Biochemistry, Michael Okpara University of Agriculture, Umudike, Nigeria; 2 Department of Biochemistry, Kogi State University, Anyigba, Nigeria; 3 Department of Biochemistry, University of Nigeria, Nsukka, Nigeria

## Abstract

**Background:**

The prevalence of hypertension, an important risk factor for cardiovascular disease (CVD), is increasing in the developing countries and this may be connected with the economic transition in those countries. Adult hypertension is thought to be related to childhood and adolescent increases in blood pressure, and hence the need to monitor patterns in early life. This study investigates the BP patterns, and their correlates, of adolescents from different geographic areas of residence in Nigeria.

**Methods:**

A total of 1,088 Nigerian adolescents from different geographic areas of residence were recruited for the study. Their blood pressures and anthropometric indices were measured using standard procedures. The association of blood pressure with height, weight, body mass index (BMI) and geographic area of residence was assessed.

**Results:**

Male and female urban-dwelling adolescents had significantly (p < 0.05) higher systolic blood pressure (117.45 ± 21.53 mmHg and 114.82 ± 17.95 mmHg respectively) compared to their counterparts living in the non-urban areas (108.20 ± 12.12 mmHg and 106.03 ± 13.06 mmHg respectively), even after adjusting for age and height. Conversely, non-urban boys (but not the girls) had significantly (p < 0.05) higher diastolic blood pressure compared to their urban counterparts. Adolescents in the urban areas had higher BMI (20.74 ± 3.27 kg/m^2 ^for males and 21.35 ± 3.37 kg/m^2 ^for females) than those in the non-urban areas (20.33 ± 3.11 kg/m^2 ^for males and 21.35 ± 3.37 kg/m^2 ^for females) though the difference was significant (p < 0.05) only in the females. Blood pressures were found to increase with age, and to be associated with BMI.

**Conclusion:**

These findings underscore the need for efforts to be made towards addressing adolescent blood pressure elevation (in both urban and non-urban areas) as they are a reflection of adult morbidity and mortality from hypertension and the associated disorders.

## Background

The greatest burden of leading communicable diseases is projected to increase substantially over the next two decades in the developing world. [1] In these countries, cardiovascular disease (CVD) is already the leading cause of mortality, [2] with mortality from ischemic heart disease expected to increase by 120% for women and 137% for men. [3] The increasing burden of CVD is largely due to the rising prevalence of many CVD risk factors, particularly hypertension. Hypertension, once rare in traditional African societies is rapidly becoming a major public health problem, [4,5] because of the increasing urbanization. [6] Socioeconomic factors may influence the distribution of high blood pressure in the developing world. However, how socioeconomic factors bring about the noticed pattern in hypertension is not clearly understood. [7] It is possible that in the less developed countries, the wealthy readily adopt unhealthy lifestyles, characterized by smoking, sedentarism and diets high in energy and fats. [8]

Hypertension in adulthood may be related to persistent blood pressure elevation in children and adolescents. [9] Assessment and management of blood pressure in childhood is therefore strongly recommended to enhance cardiovascular health in adulthood. [10] This study was carried out to provide information on blood pressure patterns and its correlates in Nigerian adolescents from three different geographic areas of residence. It is hoped that this would help in health policy formulation and the development of prevention strategies for hypertension and associated disorders.

## Methods

### Setting

Kogi State is located in the North Central geopolitical zone of Nigeria. It is so centrally located that it has boundaries with eight other states and Abuja (Nigeria's capital territory) *viz *Benue to the east, Ondo, Edo, Anambra and Enugu to the south, Kwara to the west, and Niger, Plateau and Abuja to the North. The state is bisected by the River Niger while the River Benue forms part of its northern borders. Both rivers form a confluence in Lokoja the state capital. The Igala ethnic group is the main ethnic group east of the Niger, while the Igbira and Yoruba live west of the Niger. Agriculture is the mainstay of the economy. [11] The 1991 census figure puts the population of Kogi state at 2,147,756 (1,039,484 males and 1,108,272 females). [12] However, the 2006 census preliminary results put the population of the state at 3,278,487. [13]

Lokoja (located on the west bank of the Niger River, opposite the mouth of the Benue River) is the only urban town in Kogi state. Ajaokuta (located west of the Niger, and site of an almost comatose iron and steel complex), and Ochaja (an agrarian Igala village) are two non-urban towns in the state. Two secondary schools were randomly selected, from a list of eight public schools in Lokoja, to represent the urban area. From Ajaokuta two secondary schools were randomly selected from a list of six schools, and one of the two secondary schools in Ochaja was also selected to represent the non-urban areas.

Non-urban area here refers to a town/village where some of the inhabitants are skilled or unskilled artisans, but still engage in farming and the others, say 60%, engage in subsistence farming; while urban area refers to a town with virtually all the trappings of a city – good paved roads, electricity, pipe-borne water and almost all the inhabitants do not engage in subsistence agriculture.

### Subjects

Apparently healthy secondary school children between the ages of 10 and 20 years (adolescents) who gave informed verbal consent after consulting with their parents/guardians were allowed to participate in the study.

### Methods

The study was conducted from October to December (the harvest period in Nigeria) of 2007. Data on students' ages were obtained from their school records. Age at last birthday was recorded for each student. Height was measured (with the student standing on bare feet) with a measuring tape, to the nearest 0.5 cm. Weight was measured (with the student on bare feet and with light clothing) with an electronic weighing balance, to the nearest 0.1 kg. From the heights and weights got, Body Mass Index (BMI) was calculated using the formula BMI = Weight (kg)/[Height (m)]^2^. Systolic and diastolic blood pressures were measured using an automated digital blood pressure monitor (Omron model HEM-741 CINT). The device has an error of measurement of ± 3 mmHg, according to the manufacturers. Three separate readings were taken at two minutes interval per student, with the student sitting down, and having rested for at least 10 minutes. The mean of the second and third readings was then recorded for the systolic and diastolic blood pressures respectively. The same trained personnel took blood pressure measurements in all locations. All equipments used were appropriately calibrated, according to their manufacturers' specification, before use each morning. Measurements were taken between 8 am and 10 am each day throughout the duration of the study. The Human Experiments Ethics Review Board of the Department of Biochemistry, Kogi State University, Anyigba approved the design/protocol for this study. Additional approvals were sought and obtained from the principals of participating schools.

### Data Analysis

Mean values for the different data collected in the appropriate groups were calculated and differences between means separated, with the least significant difference (LSD) fixed at 0.05. Subjects from the urban area were tagged three while those from the non-urban areas were tagged one, to reflect, numerically, the difference in both places of domicile. Pearson's product moment correlation coefficients for the variables were calculated. Multiple linear regression analysis was also done to clarify the association between geographic area of residence and blood pressures, while controlling for age and height which are confounding factors. All data analysis was done using SPSS for windows version 11.0 (SPSS Inc. Chicago, IL). Results are presented as means and standard deviations from means in tables and line graphs.

## Results

One thousand and eighty eight subjects participated in the study. Both systolic and diastolic blood pressures (SBP and DBP respectively) increased with age irrespective of sex and geographic area of residence (Table 1). For both SBP and DBP, there was no significant difference (p > 0.05) in the means of those aged 10 to 12 years compared to those aged 13 years. Subjects aged 18–20 years had significantly higher (p < 0.05) SBP and DBP mean values than the others, except those aged 16 years. However, comparing any of the ages younger than 14 years with any of those older than 14 years showed significant differences (p < 0.05) in their mean systolic but not diastolic blood pressures.

**Table 1:** Distribution of parameters measured with respect to age, but irrespective of sex and geographic area of domicile

Age (years)	Systolic BP (mmHg)	Diastolic BP (mmHg)	Weight (kg)	Height (m)	BMI (kg/m^2^)
10–12 (ma = 11.37 ± 0.68; n = 124)	102.54 ± 12.43	55.95 ± 10.78	42.32 ± 6.72	1.50 ± 0.07	18.69 ± 2.29
13 (n = 145)	103.94 ± 11.28	57.21 ± 8.18	46.16 ± 7.04	1.54 ± 0.08	19.39 ± 2.53
14 (n = 188)	108.16 ± 14.79	58.93 ± 10.03	47.93 ± 7.09	1.56 ± 0.07	19.69 ± 2.65
15 (n = 187)	111.46 ± 14.17	58.89 ± 11.22	52.10 ± 7.55	1.59 ± 0.07	20.67 ± 2.55
16 (n = 161)	115.15 ± 15.62	60.36 ± 13.09	54.75 ± 7.44	1.60 ± 0.06	21.33 ± 2.91
17 (n = 118)	112.94 ± 19.22	58.80 ± 13.46	56.96 ± 7.10	1.61 ± 0.08	22.16 ± 3.28
18 – 20 (ma = 18.47 ± 0.66; n = 165)	118.09 ± 18.32	62.79 ± 13.93	60.59 ± 8.10	1.63 ± 0.07	22.68 ± 3.79

n, ma and BP represent number of participants, mean age and blood pressure respectively

Data (mean ± standard deviation) are for systolic and diastolic blood pressures, weight, height and body mass index of all the subjects studied (1088 in number) distributed according to their ages, irrespective of sex and geographic area of residence as follows: 10–12 years (n = 124) 13 years (n = 145) 14 years (n = 188) 15 years (n = 187) 16 years (n = 161) 17 years (n = 118) 18–20 years (n = 165)

Both the heights and weights of the participants increased with age, though those aged 17 years were slightly shorter than those aged 16 years. The differences in the mean heights of subjects of any age compared with those of any other age was significant (p < 0.05) except for the differences in the mean heights of those aged 16 and 17 years. The mean weights of all the different ages differed significantly (p < 0.05) from each other. The subjects' BMI also increased with age. The increases were not however significant (p > 0.05) between those aged 13 years and 14 years, and between those aged 17 years and 18–20 years (Table 1).

**Figure 1:**
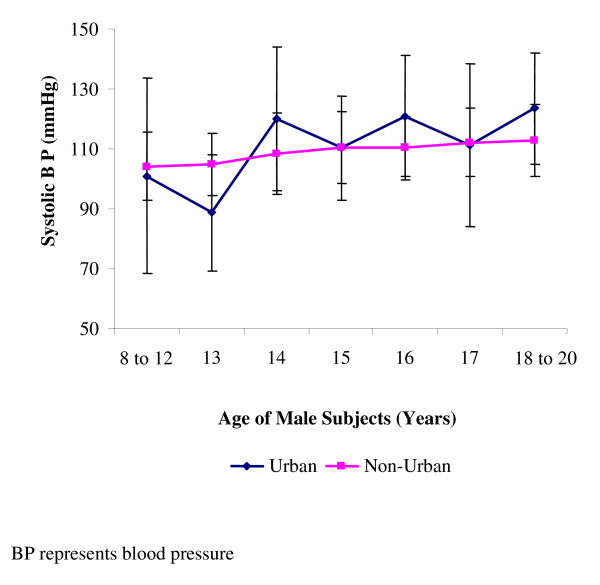
**Mean systolic blood pressures of male subjects**. Data (mean ± standard deviation) are for diastolic blood pressures of 615 males studied, and made up of 185 urban dwellers and 430 non-urban dwellers.

**Figure 2:**
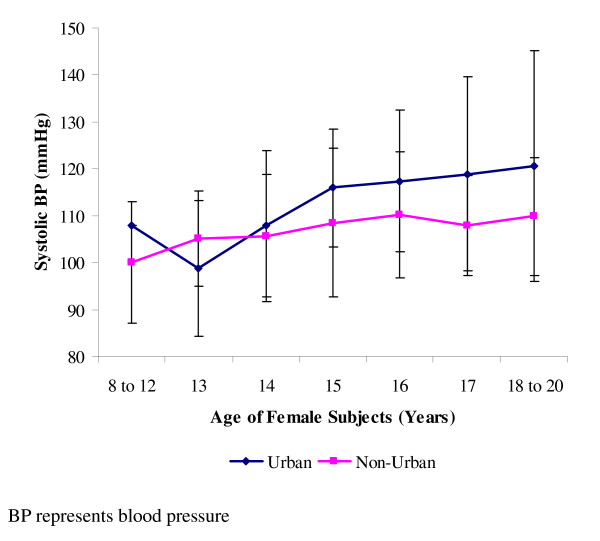
**Mean systolic blood pressures of female subjects**. Data (mean ± standard deviation) are for systolic blood pressures of 473 females studied, and made up of 217 urban dwellers and 256 non-urban dwellers.

The systolic blood pressure of the participants for both sexes increased from the non-urban dwellers to the urban dwellers (Table 2). The differences in the systolic blood pressures of the two groups were significant (p < 0.05) for both sexes. The systolic blood pressure of the urban males dropped in every other age from 10–12 years, thereby giving an ascending zigzag pattern as against the gradually ascending pattern of the non-urban group. (Figure 1). All the two residential groups however, had their male systolic blood pressures peak at 18–20 years. For the females, the systolic blood pressure of the urban dwellers dropped at 13 years, but thereafter rose gradually until it peaked at 18–20 years (Figure 2). Those of the non-urban females also peaked at 18–20 years, but were generally lower than those of the urban females.

**Table 2:** Distribution of parameters measured with respect to sex and geographic area of domicile

	Male subjects			Female subjects		
	
	Urban (n = 185)	Non-urban (n = 430)	p	Urban (n = 217)	Non-urban (n = 256)	p
Age (years)	16.72 ± 1.74	14.36 ± 2.14	<0.00	15.70 ± 1.55	14.40 ± 2.15	<0.00
Systolic BP (mmHg)						
Unadjusted	117.45 ± 21.53	108.20 ± 12.12	<0.00	114.82 ± 17.95	106.03 ± 13.06	<0.00
Age-adjusted	114.58 ± 19.21	109.65 ± 12.08	0.03	113.64 ± 17.83	105.66 ± 13.23	<0.00
Height-adjusted	115.53 ± 21.14	109.99 ± 11.94	<0.01	114.82 ± 17.95	105.60 ± 12.18	<0.00
Diastolic BP (mmHg)						
Unadjusted	55.59 ± 16.75	59.19 ± 8.67	<0.00	60.82 ± 14.59	60.18 ± 8.34	0.55
Age-adjusted	52.63 ± 16.05	59.89 ± 7.79	<0.01	60.96 ± 14.35	60.30 ± 8.32	0.60
Height-adjusted	54.72 ± 16.73	60.59 ± 8.00	<0.00	60.82 ± 14.59	59.99 ± 8.36	0.46
Weight (kg)	56.17 ± 8.54	50.27 ± 9.49	<0.00	51.30 ± 8.03	51.12 ± 9.59	0.83
Height (m)	1.63 ± 0.06	1.57 ± 0.09	<0.00	1.55 ± 0.07	1.57 ± 0.08	0.01
BMI (kg/m^2^)	20.74 ± 3.27	20.33 ± 3.11	0.14	21.35 ± 3.37	20.60 ± 2.97	0.01

n, p and BP represent number of participants, level of significance and blood pressure respectively

Data (mean ± standard deviation) are for age, systolic and diastolic blood pressures, weight, height and body mass index of all the subjects studied (1088 in number) distributed according to their sexes and geographic area of residence as follows: urban dwellers [Males (n = 185), Females (n = 217)], non-urban dwellers [Males (n = 430), Females (n = 256)]

**Figure 3:**
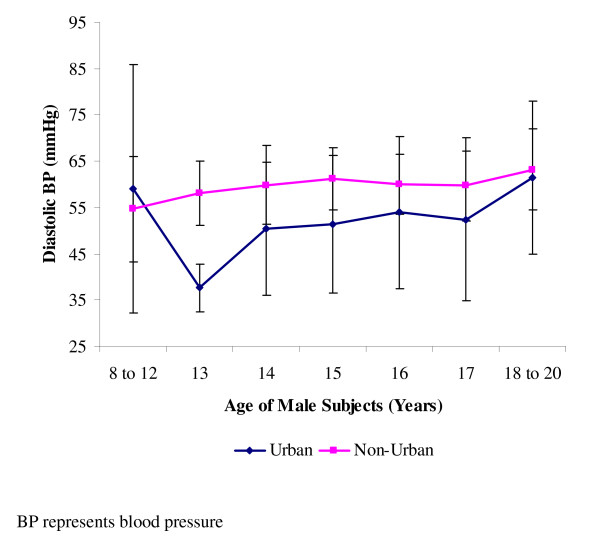
**Mean diastolic blood pressures of male subjects**. Data (mean ± standard deviation) are for diastolic blood pressures of 615 males studied, and made up of 185 urban dwellers and 430 non-urban dwellers.

**Figure 4:**
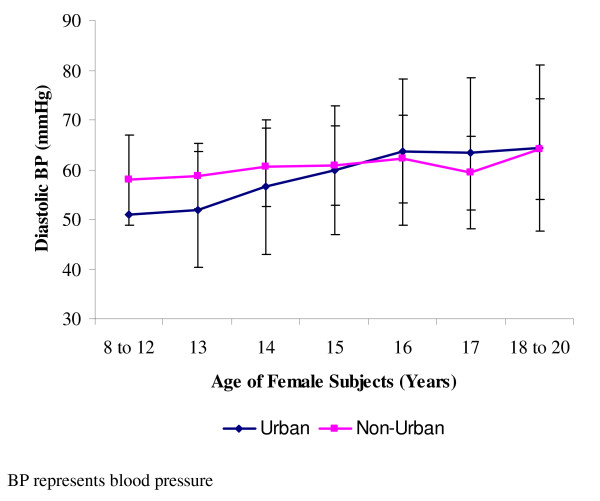
**Mean diastolic blood pressures of female subjects**. Data (mean ± standard deviation) are for systolic blood pressures of 473 females studied, and made up of 217 urban dwellers and 256 non-urban dwellers.

Diastolic blood pressures were however higher in the non-urban dwellers when compared to the urban dwellers (Table 2). The difference in the means of diastolic pressure of the males of the urban area compared to the non-urban dwellers was significant (p < 0.05). On the other hand, for the females, mean diastolic pressure differences were similar in both urban and non-urban dwellers. The diastolic blood pressures of the males of the two groups gave each, an ascending pattern, and peaked at 18–20 years (Figure 3). For the females, the diastolic blood pressures all peaked at 18–20 years, with those of the urban dwellers ascending more steeply than those of the non-urban dwellers which hovered around 60 mmHg (Figure 4).

Heights and weights were significantly (p < 0.05) higher in the urban male population compared to the non-urban male group. The non-urban girls were however, significantly (p < 0.05) taller than the urban girls. The weights of the girls in both groups were similar (Table 2).

BMI increased from 20.60 ± 2.97 kg/m^2 ^and 20.33 ± 3.11 kg/m^2 ^for the females and males respectively of the non-urban population to 21.35 ± 3.37 kg/m^2 ^and 20.74 ± 3.27 kg/m^2 ^for females and males respectively of the urban group. The difference between the mean BMI value of the females in the two groups was significant (p < 0.05). For the males however, the difference between the mean BMI scores of the urban dwellers and the non-urban dwellers was not significant (p > 0.05). (Table 2)

Correlation coefficients show that systolic blood pressure correlated positively with diastolic blood pressure (r = +0.500, p < 0.01). Both systolic and diastolic blood pressures correlated positively with age (r = +0.305, p < 0.01 and r = +0.161, p < 0.01 respectively), weight (r = +0.390, p < 0.01 and r = +0.270, p < 0.01 respectively), height (r = +0.260, p < 0.01 and r = +0.122, p < 0.01 respectively) and BMI (r = +0.265, p < 0.01 and r = +0.230, p < 0.01 respectively). However, while the correlation between diastolic blood pressure and geographic area of residence was negative and insignificant (r = -0.047, p > 0.05), systolic blood pressure correlated positively and significantly with geographic area of residence (r = +0.259, p < 0.01). The multiple linear regression analysis showed that geographic area of residence was positively and significantly (β = +0.249, p < 0.01) associated with systolic blood pressure but negatively and significantly (β = -0.210, p < 0.01) associated with diastolic blood pressure.

## Discussion

The increase in blood pressure with age found in this study is consistent with the findings of other researchers both in Nigeria and elsewhere. [14-18] This increase was not localized to any setting, though it was more prominent in the urban area. Obika *et *al [18] and Agyemang *et al *[17] had earlier reported similar findings in the blood pressures of adolescents in different populations of sub Saharan Africa. This suggests that the change in lifestyle that has been incriminated in the causation of the increasing prevalence of hypertension in sub Saharan African cities may also be creeping into the non-urban areas.

Though blood pressure generally increased with age, there were marked differences especially in the systolic blood pressures of urban dwellers when compared to non-urban dwellers. Urban dwelling adolescents had significantly (p < 0.05) higher systolic blood pressures (both for males and females) than the non-urban group. However, non-urban boys had significantly (p < 0.05) higher diastolic blood pressures when compared to those in the urban area. These differences persisted even after adjusting for age and height. The increase in diastolic blood pressure of the non-urban boys, relative to the urban boys apparently suggests a possible fading of the protection from blood pressure elevation which traditional non-urban sub-Saharan African societies once had.

Environmental factors that contribute to cases of high blood pressure, and the substantial disparity in the blood pressures across geographically different residential groups, are not well understood. [8] The relationship between geographic area of residence/socioeconomic status and blood pressure in the developing countries may be as a result of emerging new risk factors for hypertension like fetal under nutrition and psychosocial stress which may affect the poor disproportionately. [7,19] Granted that the pathway linking these variables is not clearly understood, what is worrisome is that childhood hypertension is a pointer to morbidity and mortality in adulthood. The observed higher systolic blood pressures of the urban-dwelling adolescents is even more worrisome as about 25% of Nigerians currently live in cities and the figure is expected to double in the next twenty years. [13] The burden this, coupled with the reported elevation in diastolic blood pressure in rural boys, could place on the healthcare system is enormous.

The effect of BMI on high blood pressure has been demonstrated in other populations, [17,20-23] though the exact mechanism is poorly understood. Our study showed that urban dwelling boys were significantly (p < 0.05) taller and heavier than (but had similar BMI with) their counterparts dwelling in non-urban areas. The non-urban females had similar weights with their urban counterparts, but were however, significantly (p < 0.05) taller and had significantly (p < 0.05) lower BMI compared to the urban dwelling girls. These may be related to access to food and physical exertion. Systolic and diastolic blood pressures also correlated with height, weight and BMI, positively. This trend emphasizes the role of lifestyle in increasing blood pressure, since increasing BMI is directly related to lifestyle – sedentarism, high energy and high fat diets, etc. The problem here is that these lifestyles come with urbanization, and may increase with increasing socioeconomic empowerment. However, in the developed countries, according to Mendez and colleagues, [8] the reverse is the case as high blood pressure is negatively correlated with socioeconomic status. The said authors explain that in the developed economies, high socioeconomic status groups may be the first to adopt lifestyles that lower CVD risk, or to receive better treatment, while in the less developed countries, the rich (who are almost always urban dwellers) are the ones who engage in unhealthy lifestyles.

Our study may be limited by the fact that blood pressures were measured only at one visit. We did not also assess salt intake and urinary sodium excretion in the subjects and could not lay hands on the birth weights of the children as most of them were not born in standard hospitals were appropriate records are kept. Again, since pubertal maturation occurs at different times for the two sexes (and even for people of the same sex), and is accompanied by blood pressure increases, our data should be interpreted with caution. This is because we did not assess pubertal status in the present study. Male and female subjects in the non-urban areas were significantly younger than their counterparts in the urban areas. This may be because of cultural practices that require girls to marry early, thereby dropping out of school. Boys also often drop out of school and migrate to urban areas in search of better sources of livelihood.

## Conclusion

Our results show that blood pressure increased with age in the studied population, irrespective of the geographic area of residence. SBP was significantly (p < 0.05) higher in the urban adolescents of both sexes, while DBP was significantly (p < 0.05) higher in the non-urban males only. These differences persisted after adjusting for age and height. The anthropometric parameters measured and BMI were also positively associated with increase in blood pressure in both sexes.

We recommend that more work should be done on the blood pressure of children and adolescents since they are pointers of adult blood pressure patterns. Steps should be taken to immediately address the rising trend of overweight and obesity in children and adolescents (irrespective of their place of domicile) as that would stem the rising prevalence of the associated disorders.

## Competing interests

The authors declare that they have no competing interests.

## Authors' contributions

CECCE participated in the study design, analyzed and interpreted the data and wrote the manuscript. CEU conceived and designed the study, and participated in data collection. LUSE participated in study design, and critically revised the manuscript for intellectual content. ATO participated in study design and data collection.

## Pre-publication history

The pre-publication history for this paper can be accessed here:


